# Sustaining Meaningful Patient Engagement Across the Lifecycle of Medicines: A Roadmap for Action

**DOI:** 10.1007/s43441-021-00282-z

**Published:** 2021-05-10

**Authors:** Maria Cavaller-Bellaubi, Stuart D. Faulkner, Bryan Teixeira, Mathieu Boudes, Eva Molero, Nicholas Brooke, Laura McKeaveney, Jeffrey Southerton, Maria José Vicente, Neil Bertelsen, Juan García-Burgos, Vinciane Pirard, Kirsty Reid, Elisa Ferrer

**Affiliations:** 1 EURORDIS-Rare Diseases Europe, Paris, France; 2 Radcliffe Primary Care Building, Radcliffe Observatory Quarter, Woodstock Rd, Oxford, OX2 6GG UK; 3 European AIDS Treatment Group (EATG), Düsseldorf, Germany; 4 European Patients’ Forum (EPF), Chaussée d’Etterbeek, Brussels, Belgium; 5 Teamit Research S.L., Barcelona, Spain; 6 The Synergist, Brussels, Belgium; 7 Novartis International AG, Basel, Switzerland; 8 Pfizer Inc, San Diego, CA USA; 9 Aragón Health Sciences Institute, Instituto Aragonés de Ciencias de la Salud (IACS), Zaragoza, Spain; 10 Health Technology Assessment International (HTAi)-Patient and Citizen Involvement Interest Group, Berlin, Germany; 11 European Medicines Agency (EMA), Amsterdam, The Netherlands; 12 Sanofi-Genzyme, Brussels, Belgium; 13 European Federation of Pharmaceutical Industries and Associations (EFPIA), Brussels, Belgium

**Keywords:** Patient engagement, Medicines development, Roadmap, Sustainability, Call to action

## Abstract

**Background:**

There is increased recognition that incorporating patients’ perspectives and insights into the medicines development process results in better health outcomes and benefits for all involved stakeholders. Despite the increased interest and the existence of frameworks and practical recommendations, patient engagement (PE) is not yet considered standard practice. The objective of this work was to provide a roadmap to support systematic change in all stakeholder organisations involved in medicines development across Europe, patients and patient organisations, medicines developers, academia, regulatory authorities, Health Technology Assessment bodies, payers, policy-makers and public research funders, to sustain PE practices.

**Methods:**

A mixed-methods approach was used by the EU-funded Innovative Medicines Initiative PARADIGM Consortium to co-develop the sustainability roadmap including background work to identify success factors and scenarios for sustainable PE. The roadmap development was based on the Theory of Change concept and populated with findings from (1) interviews with national/ and international institutions with the potential to increase PE uptake by other stakeholders; (2) multi-stakeholder workshops and webinars; and (3) consultations with specific stakeholder groups, Consortium members and a consultative body formed by international PE initiatives.

**Results:**

This roadmap sets strategic goals for the PE community to achieve meaningful and systematic PE through changes in the culture, processes and resources of stakeholder organisations. It brings in key PARADIGM outputs to work in a coordinated fashion with existing frameworks and mechanisms to achieve system-wide sustained PE.

**Conclusions:**

The roadmap provides a framework for all stakeholders to take collective action within their organisations and across Europe to implement PE in a sustainable manner.

**Supplementary Information:**

The online version contains supplementary material available at 10.1007/s43441-021-00282-z.

## Introduction

Incorporating patients’ perspectives and insights into the medicines development process is increasingly being recognised and accepted by all stakeholders as an important part of the process of developing innovative medicines that better address patients’ unmet needs and priorities [1–3]. Moreover, there is a drive to generate contextualised metrics [4, 5] that better capture and demonstrate the value that all stakeholders can derive from patient engagement (PE).

Patients’ and their perspectives are increasingly incorporated and valued at different stages of the medicines development process [6] including: research prioritisation (e.g. helping to define unmet needs) [7, 8]; clinical programme and clinical trial design [9–11]; early dialogue with regulators [12], health technology assessment (HTA) bodies [13] and competent authorities on pricing and reimbursement (‘payers’) [14]; as well as during the regulatory approval [15, 16] and post-approval phases including HTA evaluation [17, 18] and payer decision-making [19]. For this article, medicines development includes the above-mentioned stages. Although acceptance, implementation and regulatory expectations [20] of PE is expanding, it is not yet a standard practice and is still driven by the innovators and early adopters [21] within stakeholder groups, with little widespread urgency towards its full implementation [22].

Whilst multiple frameworks, principles and practical recommendations to operationalise and facilitate the engagement of patients exist, they are often intended for different purposes. Some are designed to facilitate PE in medicines development [9, 23–29] whilst others cover broader engagement in research or the healthcare system [6, 30].

This multitude of tools and frameworks allows each stakeholder organisation to use those that best fit their needs; however, they may be short-lived, lack transferability and hinder a consistent approach to sustaining PE practices beyond a few projects. In addition, PE is often approached superficially focusing on the individual’s choice and satisfaction rather than providing input into project design [31–33]. Failing to demonstrate that PE provides tangible benefits to all stakeholders may threaten the consolidation of PE practices [4].

The Innovative Medicines Initiative (IMI)-funded PARADIGM (Patients Active in Research And Dialogues for an Improved Generation of Medicines) Consortium (‘the Consortium’) defined PE as the effective and active collaboration of patients, patient advocates, patient representatives and/or carers in the processes and decisions within the medicines development lifecycle, along with all other relevant stakeholders when appropriate [34]. The Consortium had two main objectives: (1) to co-develop a comprehensive set of tools and practices to support the integration of patient perspectives into medicines development lifecycle and to demonstrate ‘the return on engagement’; and (2) to deliver a strategic sustainability roadmap to support systematic change in stakeholder organisations to make PE common practice. Roadmap implementation relies on the adoption and long-term use of the co-designed resources, in complementarity with existing PE frameworks, and leveraging multi-stakeholder networks. This article describes the development and contents of this strategic roadmap and calls to action to all organisations/institutions involved in medicines development, i.e. patients and patient organisations (POs), medicines developers, academia, regulatory authorities, HTA bodies, payers, policy-makers and public research funders, to achieve meaningful and sustainable PE for better health outcomes.

## Roadmap Development Process

The PE sustainability roadmap was developed by a dedicated multi-stakeholder working group within the Consortium formed by representatives of POs, medicines developers, HTA bodies and academia through a sequence of steps described in Table 1. Detailed methodology has been reported in the corresponding project deliverable [35].

**Table 1:** Summary of Methods

Sequence of methodological steps	Method type	Sources of information	Type of information sought
Step 1. To understand stakeholder needs and preferences with regards to PE, including sustainability (36)	Survey	Responses (*n* = 372) from stakeholders: patients, industry, research and academia, health care professionals, HTA bodies, regulators and research funders across Europe	Assessment of the needs and preferences of stakeholders with regards to all aspects of PE, including sustainability
Step 2. To understand how changes in the culture, processes and resources influence organisations’ sustainability (35)	Desk search and interviews (informal), benchmarking exercise	Initiatives/organisations (*n* = 19) in the open innovation field related or not with PE in medicines development. Selected initiatives based on the shared value (71) and collective impact (76) models	Identify success factors and challenges for long-term survival and operational sustainability
Step 3. To identify key elements for the development of scenarios for sustainable PE through strategic planning tools (37)	Structured brainstorming exercises: business model canvas workshop, SWOT analysis and prioritisation workshops	Multi-stakeholder groups formed by PARADIGM Consortium and PILG members that participated in four different workshops	Identify key elements to develop sustainability scenarios for the PE ecosystem. Description of sustainability scenarios for PE that responded to the questions: (1) What will drive the practice of PE and make it systematic? (2) What will reinforce the uptake of best practices? (3) How to finance PE and maintain trust, respect, and independence?
Step 4. To identify preferred scenarios for the sustainability of the PE ecosystem (38)	Consultation	Responses (*n* = 43) from PARADIGM Consortium and PILG members	Stakeholder preferences towards desired scenarios to give a high-level direction to the roadmap
Step 5. Roadmap framework development (33)	Literature search	Non-systematic review of published and grey literature. Key search words and phrases were; “sustainability roadmap”, “strategic roadmap”, “roadmap” “strategic thinking”, “uncertainty”, within the contexts of; business, science research, health, environmental sciences, sustainable enterprises, tools/toolkits to build a roadmap	The architypes of roadmaps, building a roadmap, and the underpinning common theories for strategy and roadmap creation
	Workshop	PE Sustainability Roadmap Working Group. A dedicated multi-stakeholder group within the PARADIGM Consortium that included the authors of this article	Agreement to use the Theory of Change framework to define roadmap elements (i.e. vision, mission, end and intermediate goals, actions, barriers) and how to populate them
	Interviews (informal)	Representatives from: EMA, MHRA, ICMRA, ICH. Informal discussions were moderated by members of the PARADIGM Consortium. Note: EMA and MHRA were interviewed as part of the PILG; and ICMRA and ICH for their potential to advance PE discussion and achieve broader alignment regarding PE	Capture the elements of roadmap architecture: landscape and barriers, vision, mission, long-term aspirations (end goals), intermediate goals, actions
	Survey and webinar/face-to-face meetings	Responses (*n* = 13) from PARADIGM Consortium industry partners and patient organisations’ partners (*n* = 15). Results discussed in a face-to-face meeting/online webinar with representatives from 12 industry organisations and 4 patient groups, respectively	Capture the elements of roadmap architecture: landscape and barriers, vision, mission, long-term aspirations (end goals), intermediate goals, actions
	Online workshop	PE Sustainability Roadmap Working Group	Analyse the results from interviews and surveys to populate the roadmap. Identify common long-term goals and intermediate goals and conduct a SMART (Specific Measurable Achievable Relevant Time-Bound) test to refine the goals and identify short-term changes (i.e. actions)
	Online workshop	Stakeholders involved in medicines development including patients, industry, HTA bodies, regulators from the following Central and Eastern European countries: Bulgaria, Croatia, Czech Republic, Hungary, Poland, Romania, Slovakia, Slovenia, Serbia, Latvia, Ukraine and Estonia	Test the impact and relevance of roadmap elements (intermediate goals and actions) to the CEE region

Patients were involved in the PARADIGM Consortium in all phases of the work

*PE* patient engagement, *PILG* PARADIGM International Liaison Group (a consultative body formed by international PE initiatives), *EMA* European Medicines Agency, *MHRA* Medicines and Healthcare Products Regulatory Agency, *ICMRA* International Coalition of Medicines Regulatory Authorities, *ICH* International Council for Harmonisation of Technical Requirements for Pharmaceuticals for Human Use (ICH)

The question ‘what makes PE sustainable?’—i.e. what changes are needed for PE to become common practice?—drove the initial discussions. Boutin et al. consider that, to ensure routine implementation of PE and its full integration in medicines development, it is critical to establish a culture and processes to overcome existing barriers [36]. Building on the framework proposed by Boutin et al., PARADIGM partners agreed that sustainable PE practices will also require allocation of dedicated financial and human resources, including expertise and capabilities, within stakeholder organisations. The changes needed to achieve sustainable PE were catalogued using three dimensions:

- *Culture* to drive the necessary behaviour changes to make PE common practice (‘the norm’).
- *Processes* to drive stakeholder alignment.
- *Resources* to mobilise human and financial resources towards sustained PE.

**Fig. 1:**
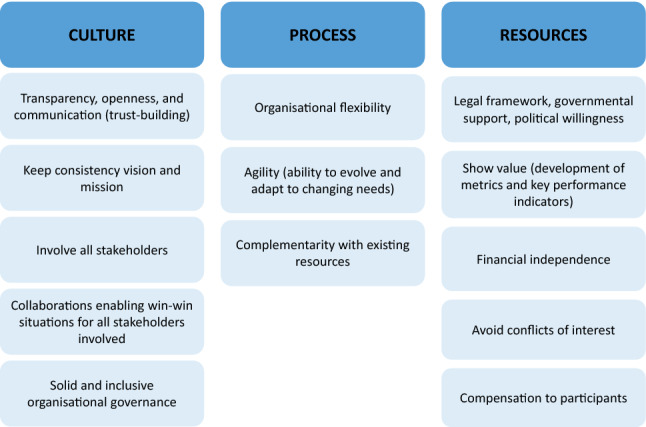
Common sustainability factors categorized according to the three sustainability dimensions. Source: Informal interviews with initiatives/organisations in the open innovation field related or not with PE in medicines development

This framework was used to conduct a broad benchmarking exercise across initiatives in the open innovation field (Table 1, step 2) that allowed for the identification of common success factors for long-term survival and operational sustainability. These factors were then categorized according to the three sustainability dimensions (Fig. 1) [37].

**Fig. 2:**
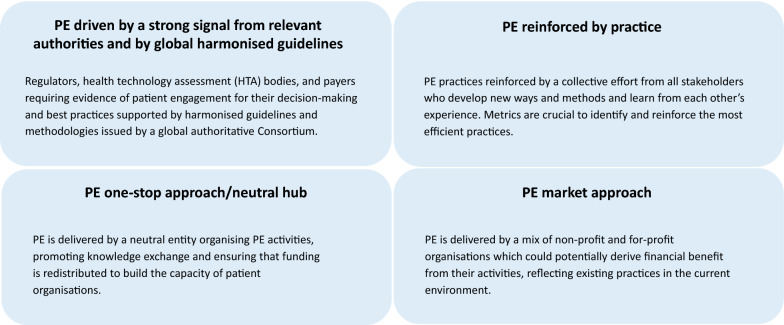
Sustainability scenarios building blocks. These components were used to describe future scenarios with the greatest potential to accelerate the implementation of PE and make it sustainable. Each of these components can drive PE within stakeholder organisations and across the medicines development ecosystem

Furthermore, the working group aimed to develop scenarios with the greatest potential to accelerate the implementation of PE and make it sustainable (see Table 1, steps 1–4). For this task, the group took into account (1) the learnings from a needs and preferences assessment (step 1) [38]; (2) the outcomes of the benchmarking exercise (step 2); (3) the outputs from the different rounds of reflection (step 3); and (4) a consultation process (step 4) [39]. Four major scenarios were suggested (Fig. 2).

For each sustainability scenario an image of how their components could concretely translate in a future PE landscape was described. Whilst PE in medicines development may continue to grow without intervention, one integrated scenario showed the potential to speed up cultural changes and adoption of new processes to sustain PE across the European region. This chosen scenario combines a strong signal from authoritative institutions to make PE a requirement in medicines development, with a diverse offering of PE services developed by for-profit and non-profit organisations [40]. PE through POs improves engagement opportunities and supports funding to further build patients’ engagement capacity, which may be at risk if POs are excluded from engagement processes. These considerations further informed development of the sustainability roadmap.

The creation of the sustainability roadmap (Table 1, step 5) [35] involved a non-systematic review of published and grey literature of models of roadmaps, building a roadmap, and the underpinning common theories for strategy and roadmap creation [41–56]. The roadmap architecture was built based on the principles of the Theory of Change model [52, 55–57] commonly used to focus the theoretical basis of a project, taking also into consideration the context in which the change will take place. Roadmaps consist of five elements that should be addressed [56, 58]:

i. Context of the initiative: analysis of current environment or landscape and actors who may influence the change.
ii. Long-term change: overall vision and desired long-term change and its expected benefits.
iii. Broad sequence of events/activities that may lead to long-term goal or change in given context.
iv. Assumptions: how change events/activities might happen and whether these activities and resulting outputs are appropriate for influencing the desired change in given context.
v. Change diagram and narrative summary.

To populate this framework, further data were gathered (see Table 1, step 5) through stakeholder group consultations within the Consortium and informal interviews with regulators and other supra-national bodies with a potential role in harmonising PE requirements and setting priorities. The roadmap was further refined for relevance of roadmap elements (intermediate goals and actions) to the Central and Eastern Europe (CEE) region.

## The Patient Engagement Sustainability Roadmap

The aim of a roadmap is to map out the desired future (not to predict it) and to provide a tool for collaborative strategic planning that enables stakeholders to make strategies and take actions towards that desired future [47].

### Context, Landscape and Barriers to PE

Although opportunities for multi-stakeholder PE collaborations have become more common, the current landscape still presents barriers, including: cultural and political barriers; lack of knowledge and experience to carry out PE; methodological barriers; barriers to PE implementation; lack of human and financial resources; confidentiality and conflict of interest; and preconceptions about PE (Table 2). Such barriers have been described elsewhere [1, 38, 59–62].

**Table 2:** Barriers to Patient Engagement as Reported by Stakeholders

Type	Description	Reported by^a^
Cultural, political	Language, cultural and political aspects of each country/region may make the adoption of PE practices difficult (e.g. disease-related stigma may be more relevant in some countries than others; changing priorities in healthcare)	PO, HTA bodies
	Fragmentation (e.g. different diseases and geographic regions, with different needs and interests, and competing for limited funding)	PO
	Lack of harmonised patient input in key policy development areas due to competing priorities between POs	PO
	English-centricity of PE practices	Regulators, HTA bodies
	Regulatory and legal environment not evolving along with PE	Medicines developers
	PE practices may be designed with a pan-European approach (e.g. engagement at EMA, global clinical development programmes)	Multi-stakeholder CEE workshop
	Building and maintaining physical and virtual platforms for discussion and exchange between organisations challenging in some countries due competing strategies of organisations	Multi-stakeholder CEE workshop
	In some countries, lack of PE and POs’ visibility, and lack of agreed communication channels between patient organisations and other stakeholders PO can have competing priorities as to where patient input is focused	Multi-stakeholder CEE workshop
Lack of knowledge, skills or experience	Lack of PE skills and limited knowledge on how to meaningfully involve patients in existing processes	POs, medicines developers, regulators, HTA bodies, ICH
	Lack of understanding of the public about their role in medicines development and the role of regulatory authorities	Regulators
	Lack of PE at the early stages of a process/project	PO
	Practices and processes not adapted to patients’ needs	PO
	Lack of understanding of medical discussions and decisions	PO
	Lack of patient leadership	PO
	Lack of health and PE literacy	PO, medicines developers
	Lack of knowledge on how to identify the right patients for the required activity	Medicines developers, HTA bodies
Methodological	Patients' needs not present	PO
	Superficial engagement with HTA bodies and payers	PO
	Lack of knowledge on how to apply methodologies to capture and use patients’ insights	Medicines developers
	Existing evidence to prove that PE leads to better health outcomes is still immature	Medicines developers
	Insufficient data to demonstrate value and impact of PE for all stakeholders and that ultimately it may not be perceived as a priority to be addressed	PO, medicines developers
	Lack of alignment across authorities on how to define and integrate a PE framework that is applicable to the local population needs and policies	Multi-stakeholder CEE workshop
	Little experience integrating and weighting patients' data vs clinical data	Regulators
Implementation of PE	Lack of accountability mechanism of patient engagement and of defined policies and practices	Medicines developers
	Lack of harmonised approach to PE	Medicines developers
	Prioritisation of PE activities where the patients’ voice adds more value vs including patients’ voice in all activities	Regulators
	Practical aspects and logistics	Regulators
	EU guidelines and frameworks not transferable at local level	PO
Lack of resources	Lack of appropriate culture and human and financial resources at organisational level	POs, medicines developers, regulators, HTA bodies
	Lack of an organisational culture supportive of PE amongst upper management	Medicines developers
	Long-term efforts and relationships required for optimal patient engagement outcomes might not align with the tight timelines of medicines development, hence risking patient engagement sustainability	Medicines developers
	Lack of financial resources to cover the expenses incurred	PO
	Funding limited to short-term projects and not ensuring long-term sustainability	PO, multi-stakeholder CEE workshop
	Lack of funding diversification	PO, multi-stakeholder CEE workshop
	The lack of continuity of patient representatives (due to disease burden or low patient numbers, such as in rare or complex diseases) results in loss of knowledge and expertise and limit the availability human resources	PO
	Lack of funding to support development of new patient advocates and leaders for long-term activism	PO
	Lack of organisational capacity may prevent from incorporating good practices	PO, medicines developers, others
Conflict of interest and confidentiality	Lack of public (government/health ministry) funding for PO	PO, multi-stakeholder CEE workshop
	Funding coming from a single source (private funding)	PO, multi-stakeholder CEE workshop
	Risk of patients’ losing their independence due to professionalization (e.g. becoming consultants)	Regulators
	Confidentiality barrier makes for lack of transparency and low PE	Regulators
Preconceptions	Preconceptions about the value of the contribution of some patient groups (such as children and young patients and people living with dementia) and the challenges of involving them	POs
	In some CEE countries there may be a perception of a lack of value of any contribution of patients or patient organisations	PO
	Lack of understanding of the value of PE	PO
	Initial mistrust from engaging stakeholders	PO
	Negative perception of industry engaging with patients	Medicines developers

^a^*Sources* Informal interviews with regulators (European Medicines Agency, UK Medicines and Healthcare Products Regulatory Agency, International Coalition of Medicines Regulatory Authorities) and the International Council for Harmonisation of Technical Requirements for Pharmaceuticals for Human Use (ICH). Internal consultations with PARADIGM Consortium patient organisations (PO) partners; PARADIGM industry partners (medicines developers). Multi-stakeholder CEE workshop on patient engagement (see Table 1 for further details)

Barriers vary according to country-level political, economic, socio-cultural, and technological contexts, e.g. some barriers are more challenging in CEE countries. For example, in some countries, socio-cultural differences in the perceived value and impact of PE by and between stakeholders (including governments and health ministries) and distrust are reported as important barriers hampering PE initiation or sustainability. Immature technological and financial infrastructure and support mechanisms for PO and PE related activities also contribute to sub-optimal PE. In general, PE and collaboration between stakeholders are more closely aligned and mature amongst Western EU Member States (MS) compared to CEE MS. Specific nuances of legal, ethical and cultural norms are often amplified across borders. Accordingly, some countries may need to address certain specific national barriers to PE before being able to tackle the wider range of goals and actions described in the roadmap (Box 1).

**Box 1:**
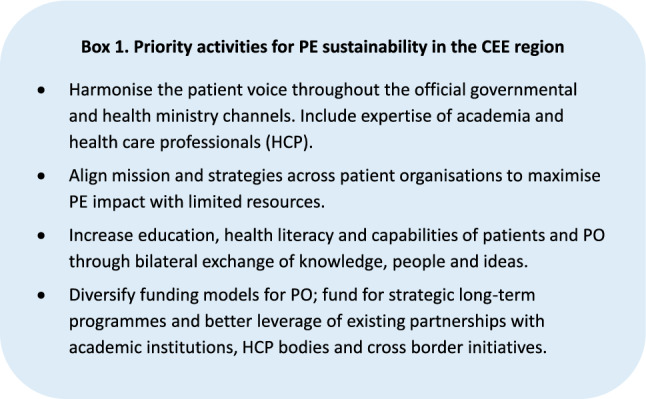
Priority activities for PE sustainability in the CEE region

### End Goals

**Fig. 3:**
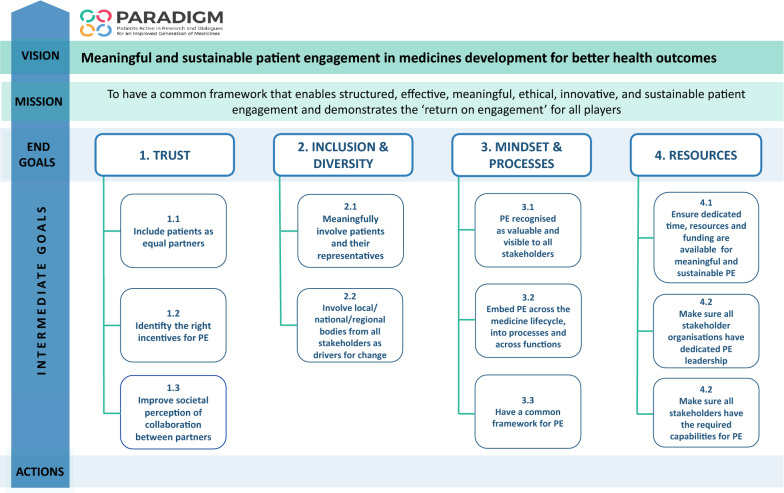
PE sustainability roadmap visual summary

The PE sustainability roadmap (33) outlined in this paper (Fig. 3) has the following elements: (a) *the vision* which defines ‘why we need to take action’, (b) *the mission* which describes ‘what needs to be done’, (c) *the end goals*, which are aspirational long-term outcomes, (d) *the intermediate goals*, which are major steps towards achieving the end goals, and (e) *the actions* needed to implement/deliver the goals and ultimately the vision.

The roadmap covers four end goals which must be met to overcome identified barriers and achieve meaningful and sustainable PE for better health outcomes:

1. Establish an ethical, trust-based collaboration amongst all PE stakeholders involved in medicines development.
2. Secure inclusive and diverse PE.
3. Embed PE in the mind-set, at every step and across organisations.
4. Ensure dedicated leadership and operational time, resources and funding for PE.

The actions described under each end goal are targeted to *all* organisations/institutions involved along the medicines development process.

#### End Goal 1: Establish an Ethical, Trust-Based Collaboration Amongst all PE Stakeholders Involved in Medicines Development

Trust is one of the critical underlying factors for sustained long-term collaboration and success. To achieve this goal, the three areas for action below were identified.

##### Include Patients as Equal Partners

The patient community (patients, carers, advocates, POs) has a key role to play in increasing its credibility and recognition by other stakeholders. Strategic alignment across POs to bring a unified voice into decision-making bodies and policy strategy is critical in general and especially so across the CEE region, where there is often a more serious disconnect between POs and decision-makers. Community and partnership-building and knowledge transfer of best practices will help define PO strategy and objectives towards PE.

Achieving an equal partnership requires a strengthened organisational capacity for systematic strategic PE within all stakeholder organisations. Demonstrated commitment from executive leadership is needed to include patient insights in decision-making processes. In addition to developing their internal capacity, it is key that stakeholder organisations also contribute to building the capacity of the patient community to meaningfully engage and interact. Other actions required include raising awareness on PE impact and practices, listening to advocacy campaigns from initiatives promoting PE and building alliances with institutions that already work with established processes of PE.

##### Identify the Right Incentives and Motivators for PE for All Stakeholders

Using a monitoring and evaluation framework to measure and demonstrate the impact of PE activities can help build the motivation, incentives and subsequent cultural shift to improve PE uptake in stakeholder organisations [4, 5]. Potential incentives could be internal to the organisation (e.g. a publicly recognised award given to staff/departments showing impact of their PE strategies and activities) or external (e.g. EURORDIS Black Pearl Awards, EFPIA Health Collaboration Award).

##### Improve the Societal Perception of Collaboration Between Patients, Their Organisations, and Other Stakeholders

Collaborations between patients, their organisations and other stakeholders need to become standard practice. Hence all parties benefit from an improved external perception towards these collaborations which may also enhance the overall willingness to engage. Supportive actions include following an established, ethical and transparent framework to help deliver PE [63], communicating on successes and failures through established and trusted platforms, being involved in public–private partnerships, and ensuring that processes are ethical, transparent and relevant between PE and other activities to proactively manage and mitigate potential conflicts of interests.

#### End Goal 2: Secure Inclusive and Diverse Patient Engagement

##### Meaningfully Involve Patients and Their Representatives

Patients’ needs and perspectives may vary widely within and across diseases, geographic regions, sociodemographic and other characteristics. It is important for all parties to acknowledge this diversity and to develop practices that recognise, respect, and incorporate the heterogeneity of patient populations and perspectives. PE in medicines development calls for the best achievable balance between diversity and the expertise and experience required for the activity. Opportunities for involvement should be maximized to incorporate a full range of patient experiences and views. Meaningful engagement could be fostered through publicly available established practices and mutually agreed objectives amongst the engaging parties where possible [64].

It is important to specifically consider requirements to engage with potentially vulnerable patient populations, patients with special needs, recently diagnosed patients and minority groups (seldom-heard patients). Involving potentially vulnerable populations is often considered a challenge and consequently these patients have historically largely been excluded from PE activities. Close relationships with POs linked to the condition experienced by these communities, or other organisations with expertise in how to involve the particularly vulnerable population, could enable their involvement in a meaningful way.

**Fig. 4:**
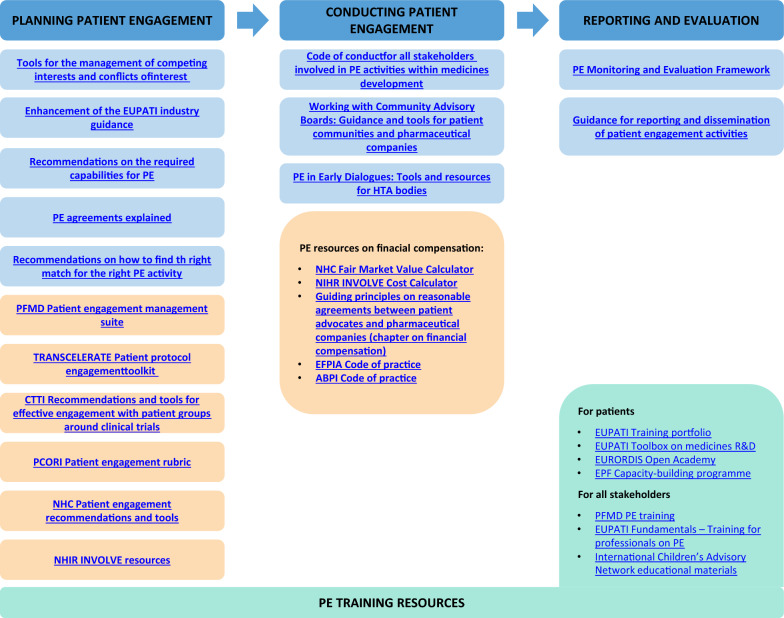
Integrated resources, guidance and tools covering PE phases. List of existing PE frameworks to achieve sustainable PE. Top headings: Phases of patient engagement. All listed resources are available and free for use and links are available in the Supplementary information. Blue boxes: Recommendations, guidance and tools included in the PARADIGM Toolbox. Orange boxes: Tools and resources from existing patient engagement frameworks (non-exhaustive). Green boxes: PE training resources (non-exhaustive). All listed resources are available and free for use. *PE* patient engagement, *PFMD* patient-focused medicines development, *CTTI* Clinical Trials Transformation Initiative, *PCORI* Patient-Centered Outcomes Research Institute, *NHC* National Health Council, *NIHR* National Institute Health Research, *EFPIA* European Federation of Pharmaceutical Industries Association, *ABPI* Association of British Pharmaceutical Industry, *EUPATI* European Patients Academy on Therapeutic Innovation, *EPF* European Patients' Forum

Organisations should raise awareness internally and externally, have policies in place on equality and diversity, as well as plan for the necessary resources, e.g. increased capacity to make the required time and accessibility adjustments and to tend to patient condition-specific needs. Integrated PE resources, guidance and tools, accompanied by training solutions (Fig. 4) provide the framework to implement ethical and meaningful PE; it will be crucial that these be disseminated to ensure widespread PE capacity-building across all parties. Capacity-building would include, but is not restricted to, informal and formal mentorship and leadership training and programmes to ensure that knowledge and expertise are built and enhanced within the broad PE community. This is particularly important in the patient community as disease burden may limit their continuity or cancel their involvement. In order to focus activities in countries and regions needing more support, a regular benchmarking exercise is recommended to assess changing attitudes to the value of PE. All stakeholders can take such actions forward in their respective organisations to prioritise resources and drive change in the areas where it is most needed.

##### Involve Local/Regional/National Bodies from All Stakeholders as Drivers for Change

PE often happens at a European or global level, e.g. European Medicines Agency (EMA), global clinical development programmes. However, local, regional and national stakeholder organisations have an important role to play as drivers for culture change in decentralised health systems. To optimise resource allocation, it is recommended to build on the existing strength of local/regional/national bodies which should utilise networking platforms to share and adopt best practices, promoting knowledge exchange in a continuous and robust manner. This can facilitate active involvement in fora for discussion and/or decision-making. This ground level approach of PE, building cooperation, sharing techniques and adopting best practices must become widespread across geographies to ensure that strategies are considered in a broader context to achieve the best possible health outcomes.

#### End Goal 3: Embed Patient Engagement in the Mind-set at Every Step and Across Organisations

##### Recognize PE as Valuable and Visible to All Stakeholders

Demonstrating and recognising the value that PE generates requires using different but complementary approaches. These include the generation of scientifically sound patient experience data through validated methodologies. This data should ideally be reported at regular intervals and analysed to provide evidence of the value added. Metrics are useful to identify how many and which type of insights provided during the PE activity have been implemented [5]. Ultimately, PE would lead to better health outcomes and therefore it is necessary to identify relevant indicators to reflect that patients’ needs are met. Identifying negative outcomes and reverse engineering them is essential to understand where and what needs to be improved.

Other actions to increase value recognition of PE involve growing a community and/or health care ecosystem that can advocate for PE (both externally and internally) and identifying champions across stakeholder groups and organisations who can articulate the risk of not engaging patients within and outside their organisations. In addition, strategic partnership of POs with higher education institutions and learned societies will be key to train the future workforce of health care organisations, industry, regulatory authorities, HTA bodies and academia on the value of PE and the role of patients in medicines development and access. A similar approach has been described elsewhere for the co-production of patient-centred health care services [65]. Public research funders and policy-makers, as well as other public health organisations and authorities, and supra-national bodies can facilitate the creation of policies and guidelines to sustain PE.

##### Embed PE Across the Medicines Lifecycle into Processes and Functions

**Fig. 5:**
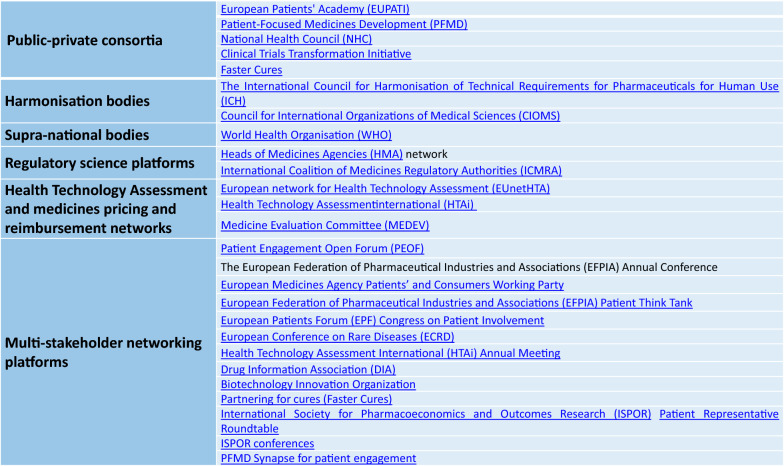
Platforms important for PE sustainability. Links to all listed resources are available in the Supplementary Information

Having a structured and consistent approach to PE processes across stakeholder organisations will help to reduce known barriers and sub-optimal PE practices. The course of actions will require that:

- Stakeholder organisations identify those points in the medicines development pathway where patients’ insights are essential and would add most value (e.g. endpoint validation, development of patient-relevant outcome measures, benefit/risk assessment, etc.).
- Stakeholder organisations define and build the required capabilities and capacities to ensure implementation of PE; rely on champions to drive culture and organisational changes; continuously learn and share best practices; and monitor the adoption of existing practices, tools and recommendations.
- Regulatory authorities, HTA bodies and payers integrate the patients’ voice into their procedures and develop a framework for engagement; set metrics to measure the degree of effectiveness of implementation of PE in their activities and processes and regularly report on their findings; ensure transparency; use existing platforms in regulatory science (Fig. 5) or other country/region-specific fora to enhance the PE discussion and ensure best practices.

##### Have a Common Regulatory Framework for PE

Regulators and policy-makers may be expected to lead the way in setting a common framework for PE [66]. Public health authorities and other supra-national bodies may help establish clear priorities for PE as part of a broader public health strategy. On the other hand, harmonised methodologies are needed to help capture patients’ insights in a systematic way that is accepted in regulatory (and HTA and payer) decision-making. The EMA Regulatory Science to 2025 has reflected on EMA’s evolving approach to patient data, emphasising the use of methodologies to collect patient experience data for benefit/risk assessment, coordinating the approach to patient-reported outcomes (PROs) and promoting the use of core health-related quality of life PROs [67]. The US Food and Drug Administration (FDA) through the Patient-Focused Drug Development (PFDD) programme [23] is developing four guidance documents for the collection and use of patient input to better inform product development and decision-making [20]. In addition, both EMA and FDA committed to formal collaboration, knowledge exchange and alignment of PE processes between the two agencies through different mechanisms [3]. In 2019, the Japanese Pharmaceuticals and Medical Devices Agency (PMDA) showed its commitment to PE by setting up a Patient Centricity Working Group to identify how patients could be involved in the Agency’s activities and to develop guidance on PE at PMDA [68]. In addition, the International Council for Harmonisation of Technical Requirements for Pharmaceuticals for Human Use (ICH) could play a significant role in the harmonisation of PE practices in clinical trial programmes. ICH has started drafting a new version of ICH E8 General Considerations for Clinical Trials guideline, which includes general considerations on patient input into study design [69]. ICH will commit to ensure the upfront involvement of all parties in study planning [70]. The Council for International Organizations of Medical Sciences (CIOMS) created in 2018 the CIOMS Working Group XI on Patient Involvement and is now elaborating a future global guidance on patient involvement [71]. “Patient participation in the generation and utilization of safety and effectiveness data” will be one of the areas covered in this guidance. These concerted efforts will no doubt lead to more effective PE and generate solid evidence for regulatory, HTA and payer decision-making.

#### End Goal 4: Ensure Dedicated Leadership and Operational Time, Resources and Funding for Patient Engagement

##### Ensure Dedicated Time, Resources and Funding are Available for Meaningful and Sustainable PE

Conducting optimal PE takes substantial resources, either in terms of operational time to carry out and deliver PE, increasing resources and augmenting staff competencies and departmental capacity, or securing continued funding for PE activities. Given the number and systematic activities proposed in the roadmap, meaningful and sustainable PE is unlikely to be achieved at current resource levels across stakeholder groups and organisations, hence human and financial resources will most likely need to be increased.

Although the industry is showing strong signs of progress in PE, the budgets, funding and resource allocation to PE units are not comparable to those dedicated for the engagement of other stakeholders (e.g. health care professionals and policy groups). There is a lack of funding for internal PE activities and insufficient internal understanding of how grants, donations and sponsorships are allocated. Industry stakeholders must continue to elevate the discussion to the highest levels on the role and competencies required for PE.

Across Europe different financial models that support PE activities will likely be needed to ensure long-term sustainability of strategies and actions. Relevant financial resources in the CEE region are currently very limited and their use is mainly reactive which undermines sustainable responses. In the case of CEE POs, funding may come from a single source (e.g. private funding), resulting in a perception of lack of PO independence. In addition, such funding is usually aimed at short-term projects rather than strategic, long-term engagements that are designed to ensure stable and sustainable responses. This might be aggravated by funding diverted to other causes in the event of global health crises (see Limitations). Demonstrating the return on engagement may help to leverage more strategic funding.

Stakeholder organisations should assess their needs for the time, personnel and funding required to conduct meaningful PE and ensure that they are addressed where it is most relevant and/or likely to be of significant impact and benefit. Depending on the type of stakeholder, other actions could involve seeking alternative funding models through national and cross-border initiatives that can promote PE processes and practices. A preferred model is one by which the financial independence of POs is secured. There can be no meaningful PE if the implementation of a compensation framework for patient participants undermines patient community participation or results in feelings of disrespect or mistrust. It is therefore critical that compensation is paid according to fair market value standards and compliant with local laws and regulations. It is also required to ensure that participation in PE activities does not place a financial burden on patients.

##### Make Sure All Stakeholder Organisations have Dedicated PE Leadership and the Required Capabilities

Regarding human resources, it is crucial to ensure dedicated PE leaders who can drive culture and organisational changes and also to equip the organisations with functions holding the required core competencies, processes, tools and systems to implement PE [72].

### Resources and Tools for Sustainability

The Consortium developed a toolbox [73] (Fig. 4) following a process of needs assessment, gap analysis and co-production to increase stakeholders’ preparedness for PE. The toolbox was not created in a vacuum but built on the back of global initiatives (e.g. TransCelerate, CTTI, NHC, PCORI, PFMD, EUPATI) and the experience of Consortium partners, and provides guidance and practical recommendations along the different phases of PE. Planning for sustainability involves knowing the available tools and using them at the right stages to ensure consistent PE practices. The implementation of the actions proposed in the roadmap will be facilitated using such tools and will result in enhancement and optimization of existing processes within organisations. The organisations within the PARADIGM Consortium are already implementing this toolbox in their activities and are highly committed to disseminate it and encourage their use within their respective constituencies and beyond. In addition, the Patient-Focused Medicines Development (PFMD) Consortium and the EUPATI Initiative will host the PARADIGM toolbox in their respective sites hence ensuring that PARADIGM outputs are widely available to stakeholder organisations beyond the Consortium [74].

Sharing the knowledge and experiences of doing PE, reaching out to stakeholders for alignment and validation, and assessing the level of institutionalisation of PE practices are essential mechanisms of a system-wide strategy to sustain PE in medicines development. Evaluation ensures alignment of the activities with the proposed framework and enables the making of necessary adaptations to reflect new knowledge and insights. Figure 5 features established public–private consortia that promote, support and develop tools for PE; harmonisation and supra-national bodies with a potential role in harmonising PE requirements and setting priorities; regulatory science, HTA bodies and payers’ networks that could enhance the PE discussion around the requirement of patient experience data in their decision-making; and multi-stakeholder networking platforms where stakeholder alignment and trust-building, together with cross-pollination of experiences and best practices, take place.

### Role of Stakeholder Organisations

The change process towards sustained PE is expected to occur at individual, organisational and systemic levels, and may happen at a different pace across geographies and organisations. The roadmap does not propose a linear timeline for actions to unfold, but instead actions can occur at non-linear checkpoints as described elsewhere [48]. This non-linear approach proposes that in the short term, organisational strategy may be driven by tactics, which are underpinned by available data, evidence and certainty. Moving beyond this to the mid- and long-term, organisations must accept some uncertainty as they continuously reflect on their learnings and recalibrate their strategy and tactics to their new starting point for systems level evolution.

When confronted with mixed urgent and important priorities (e.g. setting up collaborations to develop treatments for COVID-19 vs setting up an organisation-wide PE training), organisations risk falling into the ‘mere-urgency trap’ and focus efforts in short-term activities that may prevent them from fulfilling long-term goals [22]. Whilst the importance of patient-centeredness and PE is now beyond question for most stakeholders, achieving the roadmap’s vision will require reassessing the priority given to PE.

The implementation of the actions described in the roadmap is underpinned by two fundamental mechanisms: (1) the use of integrated PE resources and toolkits that can help all stakeholders to manage existing experiences and resources and optimize them, i.e., “to not reinvent the wheel” (Fig. 4); and (2) knowledge sharing and benchmarking within and between organisations (Fig. 5). It is essential that organisations encourage culture and process change through education and knowledge sharing of best practices internally and externally and develop the competency to measure and report on the impact of PE, including risks and impact of not doing it. External engagement will be crucial in helping to drive internal change.

The roadmap architecture and assumptions have been built based on the principles of the Theory of Change to propose a set of actions, the connections between them and the desired long-term change (i.e., end goals and vision) [58]. Monitoring progress of the implementation of the actions is necessary to redefine or update the strategy according to the evidence gathered at given checkpoints. Each stakeholder organisation may already be measuring implementation of PE practices internally and could follow similar, albeit different, methods to monitor roadmap adoption. Collective responsibility and collaborations within and across stakeholder groups and organisations will be crucial to drive the actions forward. Benchmarking mechanisms could take many forms such as open dialogues and multi-lateral exchanges of knowledge, ideas and good practices. External engagement through multi-stakeholder platforms will be key to monitor if and how changes are implemented across organisations and stakeholder groups. The roadmap encourages stakeholders to make the most of existing initiatives and networking platforms for knowledge diffusion and experience exchange, but also as mechanisms to benchmark progress towards the vision (Fig. 5). The emergence of the IMI-PARADIGM Patient Engagement Open Forum (PEOF) [75], with its hands-on approach to advance PE, could also strengthen multi-stakeholder collaborations and the collective evolution of the PE community. Former PEOF organisers (i.e. EUPATI, PFMD) together with the European Patients’ Forum reiterated their commitment and joined efforts to continue organising future editions of the PEOF following PARADIGM recommendations [74].

### Limitations

The roadmap is intended to be aspirational and envisions that PE in medicines development is sustainable, i.e. that PE as ‘business as usual’ is achievable. Despite a growing shared culture, stakeholders hold different expectations and objectives regarding PE in medicines development and therefore might have different attitudes towards the need for and impact of change [66]. We acknowledge that top-down strategies (e.g. regulators requiring evidence of PE as part of a medicine registration dossier) may help accelerate change (e.g. patient input in clinical programme).

Although the actions are intended to be taken forward by all stakeholders involved, it is acknowledged that there is no single entity in Europe responsible for implementing or updating the roadmap. As a result, there will be a need to adjust the roadmap depending on differing and possibly competing remits, processes or other influences. In some countries, it will be important to address existing barriers before its implementation (Box 1). Since periodic benchmarking of progress is currently done separately by each stakeholder using different mechanisms, it will not be easy to arrive at realistic and shared benchmarks for the roadmap across Europe. Informal collaborations amongst stakeholder groups through knowledge-sharing platforms and other mechanisms focused on PE will play a role in advancing the strategy (Fig. 5).

The roadmap has not been stress tested within any entity or stakeholder to identify possible impediments to its implementation. Soft power is relied upon to implement the roadmap and thus some elements may not be practical or feasible to implement by one or more stakeholders at any given step. The assumptions that underpin the current PE landscape and desired future state have a degree of uncertainty and limited accuracy, and therefore the roadmap may well require updating in the mid-to long-term.

The roadmap might be affected by global health crises which have an impact on countries’ political stability and the availability of resources in health care systems. PE may be particularly reduced in such circumstances, as observed during the COVID-19 pandemic [76]. POs have experienced operational difficulties and shifting priorities due to the increased demand to support their community [77], which has led to reducing other activities such as fundraising or involvement in PE activities. Nevertheless, public health emergencies may also bring opportunities for increased collaboration in which patient- and citizen-driven change may become more relevant [76–78]. The expansion of virtual events and platforms during the pandemic has increased patients’ accessibility to these events, thus facilitating discussion and exchange between patients and other stakeholders in an unprecedented manner.

## Conclusion: Call to Action

Benchmarking progress and evolving strategies are not the final steps; sustaining change requires a process of continued learning and improvement, experience sharing and production of more change (if needed) [52]. Stakeholders have now a roadmap that can be adopted and adapted within their own organisations depending on the maturity of their own PE practices. The roadmap recommends taking a series of actions towards four key end goals to ensure that PE is sustained through (1) mutual trust across stakeholder groups; (2) inclusive and diverse PE, with the involvement of organisations at all levels (not only central/global); (3) culture drivers to embed PE within and across organisations; and (4) dedicated human and financial resources. This roadmap can be regarded as a succession of interconnected milestones that are achievable thanks to the existing resources. IMI-PARADIGM has delivered a robust framework, that in synergy with others (Fig. 4), will help implementing PE processes and consolidating existing ones within individual organisations, thus fostering PE and making it sustainable. PARADIGM has also contributed to strengthen organisations’ PE readiness not only by providing actionable tools, but by bringing stakeholders together and breaking down fragmentation in the medicines development ecosystem.

However, widespread adoption and implementation of the roadmap relies on a PE community open to honest collaboration and exchange beyond their own organisation and stakeholder group to sustain change across the medicines development ecosystem. Now more than ever, sustainable PE seems within reach if all stakeholders take joint and determined action.

**Box 2:**
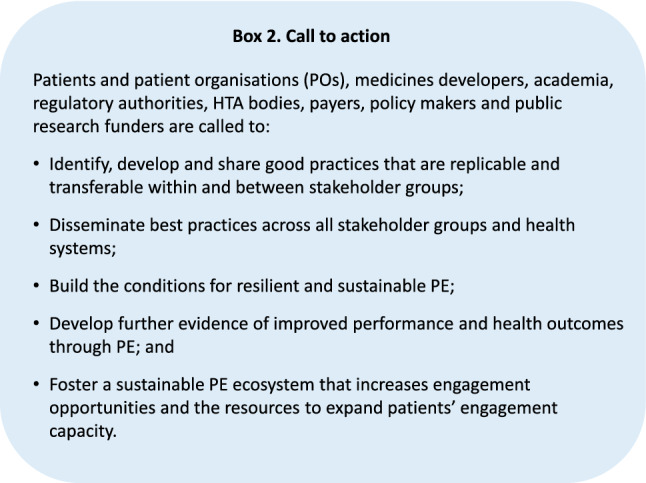
Call to action to the PE community

## Supplementary Information

Below is the link to the electronic supplementary material.

Supplementary file1 (PDF 173 kb)
