# Supplementary material for: Sustaining Meaningful Patient Engagement Across the Lifecycle of Medicines: A Roadmap for Action
Source: Ther Innov Regul Sci. 2021 May 10;55(5):936–53. doi: 10.1007/s43441-021-00282-z (PMC8108434; doi:10.1007/s43441-021-00282-z)
Supplement: Supplementary file 1 — Supplementary file1 (PDF 173 kb) [file 43441_2021_282_MOESM1_ESM.pdf]

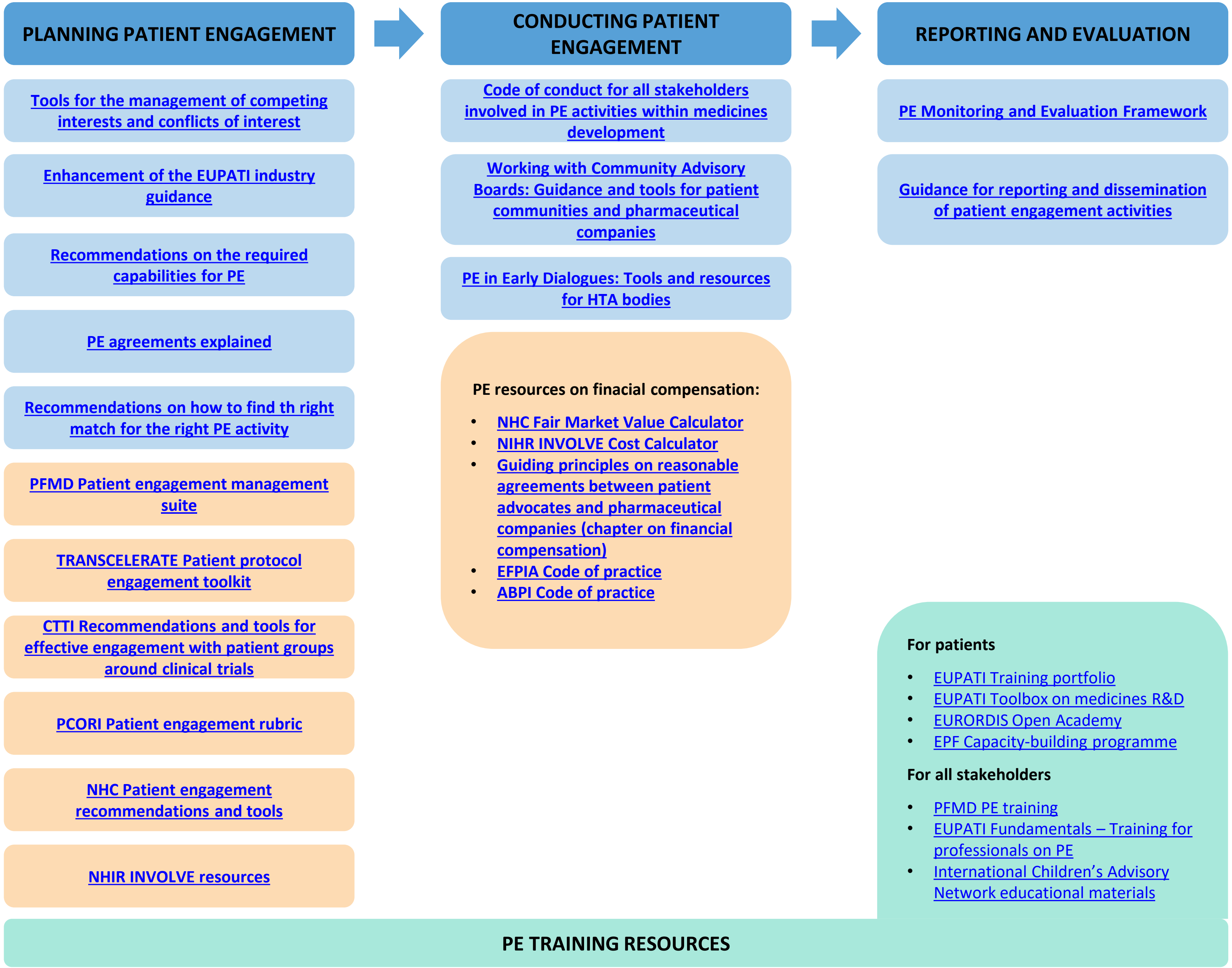

Fig. 4 Integrated resources, guidance and tools covering PE phases

**Public-private consortia**	European Patients' Academy (EUPATI)
	Patient-Focused Medicines Development (PFMD)
	National Health Council (NHC)
	Clinical Trials Transformation Initiative
	Faster Cures
**Harmonisation bodies**	The International Council for Harmonisation of Technical Requirements for Pharmaceuticals for Human Use (ICH)
	Council for International Organizations of Medical Sciences (CIOMS)
**Supra-national bodies**	World Health Organisation (WHO)
**Regulatory science platforms**	Heads of Medicines Agencies (HMA) network
	International Coalition of Medicines Regulatory Authorities (ICMRA)
**Health Technology Assessment and medicines pricing and reimbursement networks**	European network for Health Technology Assessment (EUnetHTA)
	Health Technology Assessment international (HTAi)
	Medicine Evaluation Committee (MEDEV)
**Multi-stakeholder networking platforms**	Patient Engagement Open Forum (PEOF)
	The European Federation of Pharmaceutical Industries and Associations (EFPIA) Annual Conference
	European Medicines Agency Patients' and Consumers Working Party
	European Federation of Pharmaceutical Industries and Associations (EFPIA) Patient Think Tank
	European Patients Forum (EPF) Congress on Patient Involvement
	European Conference on Rare Diseases (ECRD)
	Health Technology Assessment International (HTAi) Annual Meeting
	Drug Information Association (DIA)
	Biotechnology Innovation Organization
	Partnering for cures (Faster Cures)
	International Society for Pharmacoeconomics and Outcomes Research (ISPOR) Patient Representative Roundtable
	ISPOR conferences
	PFMD Synapse for patient engagement

Fig. 5 Platforms important for PE sustainability
